# Schizophrenia: Four new hypotheses

**DOI:** 10.1192/j.eurpsy.2021.2109

**Published:** 2021-08-13

**Authors:** B. Jorge, C. Pedro Fernandes, M. Mangas, J. Carvalho

**Affiliations:** 1 Serviço De Psiquiatria, Hospital de Braga, Braga, Portugal; 2 Psychiatry, Hospital de Braga, Braga, Portugal; 3 Serviço De Psiquiatria, Unidade de Saúde Local do Baixo Alentejo, Beja, Portugal

**Keywords:** schizophrénia, Hypothesis

## Abstract

**Introduction:**

Schizophrenia is a chronic and debilitating psychiatric disorder. Affecting social, emotional, perceptive, and cognitive domains, its clinical phenotype can be subdivided into positive and negative symptoms, and those of cognitive impairment. As the knowledge base behind the social and environmental origins accumulates, the etiological and neuropathophysiological mechanisms behind them remain elusive.

**Objectives:**

To review the latest developments in potential etiological hypotheses linked to schizophrenia.

**Methods:**

A non-systematic review was performed, searching Pubmed for articles published between the years of 2019 and 2020.

**Results:**

(1) Common genetic variants alter brain glycosylation and may play a fundamental role in the development of schizophrenia. The strongest coding variant in schizophrenia is a missense mutation in the manganese transporter SLC39A8, which is associated with altered glycosylation patterns in humans, resulting in modification of a subset of schizophrenia-associated proteins. (2) Failure of oligodendrocytes and astrocytes to differentiate contributes to several of the key characteristics of schizophrenia, including hypomyelination and abnormalities in glutamate and potassium homoeostasis. (3) Diglossia was hypothesized as a risk factor, as it could constitute a neurodevelopmental insult. This relationship may be mediated by the reduced lateralization of language in the brain. (4) The first brain-wide resting state effective-connectivity neuroimaging analysis proposed going beyond the disconnectivity hypothesis, drawing attention to differences between back projections and forward connections, with the backward connections from the precuneus and posterior cingulate cortex implicated in memory stronger in schizophrenia.

**Conclusions:**

These novel insights may be a promising step in the right direction, presenting not only new approaches towards the complex pathogenesis of schizophrenia, but also eventual early interventions.

**Disclosure:**

No significant relationships.

